# Sickle cell disease‐associated arrhythmias and in‐hospital outcomes: Insights from the National Inpatient Sample

**DOI:** 10.1002/joa3.12418

**Published:** 2020-08-08

**Authors:** Upenkumar Patel, Rupak Desai, Bishoy Hanna, Dhruval Patel, Shahzad Akbar, Mohammed Zubair, Gautam Kumar, Rajesh Sachdeva

**Affiliations:** ^1^ Department of Internal Medicine Nassau University Medical Center East Meadow NY USA; ^2^ Division of Cardiology Atlanta VA Medical Center Decatur GA USA; ^3^ Division of Cardiology Morehouse School of Medicine Atlanta GA USA; ^4^ AMC MET Medical College L.G Hospital Ahmedabad Gujarat India; ^5^ Division of Cardiology Emory University School of Medicine Atlanta GA USA; ^6^ Division of Cardiology Medical College of Georgia Augusta GA USA

**Keywords:** arrhythmia, atrial fibrillation, mortality, prevalence, sickle cell anemia, sickle cell disease

## Abstract

**Background:**

The frequency and temporal trend in the prevalence of arrhythmias and associated in‐hospital outcomes in patients with sickle cell disease (SCD) have never been quantified.

**Methods:**

Our study cohort of SCD patients and sub‐types of arrhythmias were derived from the 2010‐2014 National Inpatient Sample using relevant diagnostic codes. The frequency and trends of arrhythmia and odds of inpatient mortality were measured.

**Results:**

A total of 891 450 hospitalized SCD patients were identified, of which, 55 616 (6.2%) patients experienced arrhythmias. The SCD cohort with arrhythmia demonstrated higher all‐cause mortality (2.7% vs 0.4%; adjusted OR 2.53, 95% CI 2.15‐2.97, *P* < .001), prolonged hospital stays (6.9 vs 5.0 days) and higher hospital charges ($53 871 vs $30 905) relative to those without arrhythmias (*P* < .001).The frequency of supraventricular arrhythmia (AFib, SVT, and AF) and ventricular arrhythmia (VFib and VT) were 1893 and 362 per 100 000 SCD‐related admissions, respectively. Unspecified arrhythmias (4126) were seen most frequently followed by AFib (1622) per 100 000 SCD‐related admissions. From 2010 to 2014, the frequency of any arrhythmias and atrial fibrillation in hospitalized SCD patients relatively increased by 29.6% and 38.5%, respectively. There was nearly a twofold (2.4% in 2010 to 5.0% in 2014) increase in the frequency of arrhythmia among patients aged <18 years. The frequency of arrhythmias in hospitalized male and female SCD patients relatively increased by 28.8% and 31.4%, respectively (*P*_trend_ < .001).

**Conclusions:**

The frequency of arrhythmias among SCD patients is on the rise with worse hospitalization outcomes, including higher in‐hospital mortality and higher resource utilization as compared to those without arrhythmias.

## Graphical abstract

Nearly 6% of all SCD‐related admissions revealed concomitant arrhythmias. Rising trends in arrhythmia were seen in SCD‐related admissions from 2010‐2014. Unspecified arrhythmias were most prevalent followed by AFib. Arrhythmia events raised the odds (OR 2.53) of inpatient mortality in SCD. Length of stay and charges were higher in the SCD cohort with arrhythmia.

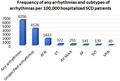

## 1 INTRODUCTION

Sickle cell disease (SCD) is one of the most common structural genetic disorders of hemoglobin, affecting around 100 000 patients in the United States (US),^1^ most often African Americans.^2^ Patients with SCD suffer from chronic hemolytic anemia and recurrent episodes of ischemia‐reperfusion injury. Among the most common clinical manifestations of SCD are vaso‐occlusive crises, which often result in recurrent hospitalizations and are an indicator of poor prognosis.^3^ The improved survival of patients with SCD owing to recent advances in treatments has resulted in a rise in the incidence of the more chronic cardiopulmonary manifestations of the disease, such as myocardial infarction (MI), pulmonary hypertension (PH), left ventricular diastolic dysfunction and cardiac arrhythmias.^4^ The frequency of sudden death in the aging SCD patient population is also on the rise.^4^, ^5^, ^6^ An investigation of an autopsy series uncovered that cardiopulmonary etiologies such as MI, PH, heart failure, and cardiac arrhythmias were the most common causes of death.^5^, ^7^ Electrocardiographic abnormalities, for example, QT prolongation and ventricular arrhythmias are not inconsistent in SCD patients.^8^, ^9^ The cumulative frequency and temporal trends of cardiac arrhythmias in patients with SCD have not been explored in the SCD population through large‐scale, cross‐sectional studies. We aimed to quantify the frequency and characterize the temporal trends of fatal and non‐fatal in‐hospital arrhythmias and consequent in‐hospital outcomes in patients primarily admitted for SCD using a nationally representative US cohort.

## 2 METHODS

### 2.1 Data source

The study cohort was acquired from 2010 to 2014 National Inpatient Sample (NIS) database, a part of Healthcare Cost and Utilization Project (HCUP), funded by the Agency for Healthcare Research and Quality (AHRQ), to evaluate the frequency and hospitalization outcomes of arrhythmias in hospitalized SCD patients. The NIS is the largest all‐payer publicly accessible inpatient healthcare database in the United States. It includes more than 7 million unweighted and more than 35 million weighted hospitalizations (national estimates) each year. Institutional review board (IRB) approval was not required as the NIS is a de‐identified database.^10^

### 2.2 Study population

International Classification of Diseases, Ninth Revision, Clinical Modification (ICD‐9‐CM) codes were used as reported earlier to identify hospitalized SCD patients as primary diagnosis (282.41, 282.42, 282.6, 282.60‐282.64, 282.68, and 282.69) and arrhythmias as secondary diagnosis (ICD‐9‐CM codes for atrial fibrillation [AFib] 427.31, atrial flutter [AF] 427.32, supraventricular tachycardia [SVT] 427.0, ventricular fibrillation [VFib] 427.41, ventricular tachycardia [VT] 427.1, atrioventricular [AV] block 426.0, 426.10, 426.11, 426.12, 426.13 and unspecified arrhythmias 427.2, 427.9, 427.89, 785.0).^3^, ^11^, ^12^, ^13^, ^14^. Discharges related to sickle cell trait (282.5) or thalassemia (282.49) were omitted from the study cohort.

### 2.3 Statistical analyses

The Pearson chi‐square test and Student's *t* test were used to assessing categorical and continuous variables, respectively. The trends in the frequency of arrhythmias in hospitalized SCD patients were measured by the linear‐by‐linear association test. After adjusting for baseline characteristics and comorbidities, a two‐step hierarchical multivariate regression model was utilized to estimate the risk of in‐patient mortality due to any arrhythmias. A multivariate regression model was adjusted for baseline demographics including age, gender, and race, payer status, admission day (weekend or weekdays), hospital bed size, location/teaching status and region, all baseline comorbidities. SPSS version 22 (IBM Corp) was used for all statistical analyses.

## 3 RESULTS

### 3.1 Study population and baseline characteristics

A total of 891 450 weighted SCD‐related hospitalizations were identified from 2010 to 2014. Of these, 55 616 (6.2%) encounters (mean age 39.55 (±20.41) yrs, 59.7% females) were associated with arrhythmias. Among hospitalized patients with SCD, the frequency of arrhythmias was highest in those aged 18‐44 years. The SCD‐arrhythmia cohort consisted more often of African Americans (88.3%) and Medicaid enrollees (36.4%) with weekday (76.1%) admissions. The SCD cohort with arrhythmia was more likely managed at large (65.6%), nonprofit private (74.2%), urban‐teaching hospital (74.5%) in the southern (49.6%) part of the US. The SCD cohort with arrhythmia had a higher prevalence of major comorbidities as compared to those without arrhythmia. The SCD cohort with arrhythmia had worse hospitalization outcomes including increased mortality (2.7% vs 0.4%), a longer length of stay (6.9 (±8.2) vs 5.0 (±6.8) days) and higher total hospital charges ($53 871 vs $30 905) as compared to hospitalized SCD patients without arrhythmias (*P* < .001) (Table 1).

**TABLE 1:** Baseline characteristics and outcomes of sickle cell disease (SCD) inpatient encounters with vs without arrhythmia(s)

Variable	SCD + no arrhythmia	SCD + any arrhythmia	*P*‐value*
Weighted N	835 834	55 616	
Age (y) at admission			
Mean age ± SD	29.0 ± 15.8	39.6 ± 20.4	<.001
<18	19.1%	11.0%	
18‐44	65.1%	49.4%	
45‐64	13.3%	27.2%	
≥65	2.5%	12.3%	
Gender			<.001
Male	37.7%	40.3%	
Female	62.3%	59.7%	
Race			<.001
White	2.4%	4.5%	
African American	90.3%	88.3%	
Hispanic	4.2%	4.0%	
Asian or Pacific Islander	0.4%	0.5%	
Native American	0.1%	0.1%	
Other	2.5%	2.6%	
Primary expected payer			<.001
Medicare	21.8%	34.8%	
Medicaid	49.3%	36.4%	
Private including HMO	21.3%	21.1%	
Self‐pay	4.4%	4.8%	
No charge	0.4%	0.3%	
Others	2.7%	2.6%	
Admission day			
Weekday	76.6%	76.1%	
Weekend	23.4%	23.9%	
Bed size of hospital			<.001
Small	10.4%	10.7%	
Medium	25.2%	23.6%	
Large	64.4%	65.6%	
Location/teaching status of hospital			<.001
Rural	4.3%	3.9%	
Urban—non‐teaching	21.7%	21.6%	
Urban—teaching	74.0%	74.5%	
Region of hospital			<.001
Northeast	22.4%	20.3%	
Midwest	19.2%	21.4%	
South	50.3%	49.6%	
West	8.1%	8.8%	
Control/ownership of hospital			<.001
Government, non‐federal	16.7%	16.2%	
Private, not‐profit	73.1%	74.2%	
Private, invest‐own	10.2%	9.6%	
Comorbidities			
Alcohol abuse	1.4%	2.5%	<.001
Deficiency anemias	8.5%	17.6%	<.001
Congestive heart failure	3.1%	12.5%	<.001
Chronic pulmonary disease	17.2%	20.8%	<.001
Coagulopathy	3.8%	8.1%	<.001
Depression	6.8%	8.6%	<.001
Diabetes, uncomplicated	4.9%	11.0%	<.001
Diabetes, chronic complications	1.3%	3.0%	<.001
Drug abuse	7.3%	7.6%	.053
Hypertension	18.5%	39.0%	<.001
Hypothyroidism	2.1%	5.0%	<.001
Liver disease	2.0%	4.2%	<.001
Fluid and electrolytes disorders	16.7%	32.4%	<.001
Obesity	6.3%	9.6%	<.001
Paralysis	1.4%	2.8%	<.001
Peripheral vascular disorders	0.8%	3.1%	<.001
Psychoses	3.1%	4.3%	<.001
Pulmonary circulation disorders	4.1%	10.5%	<.001
Renal failure	5.6%	17.0%	<.001
Valvular disease	1.4%	6.1%	<.001
Weight loss	1.6%	4.7%	<.001
Sepsis	4.1%	8.6%	<.001
Acute myocardial infarction	0.3%	1.5%	<.001
Pneumonia	8.6%	15.0%	<.001
Acute chest syndrome	5.1%	7.9%	<.001
SCD with crisis	56.5%	47.9%	<.001
Splenic sequestration	0.8%	1.4%	<.001
Outcomes			
All‐cause in‐hospital mortality	0.4%	2.7%	<.001
Total hospital charges (mean)	$30 905	$53 871	<.001
Length of stay (d) (mean ± SD)	5.0 ± 6.8	6.9 ± 8.2	<.001

Abbreviation: SCD, sickle cell disease.

* Significant *P*‐values ≤ .05 at 95% confidence interval.

### 3.2 Frequency and trends in arrhythmias among SCD‐related hospitalizations

**FIGURE 1:**
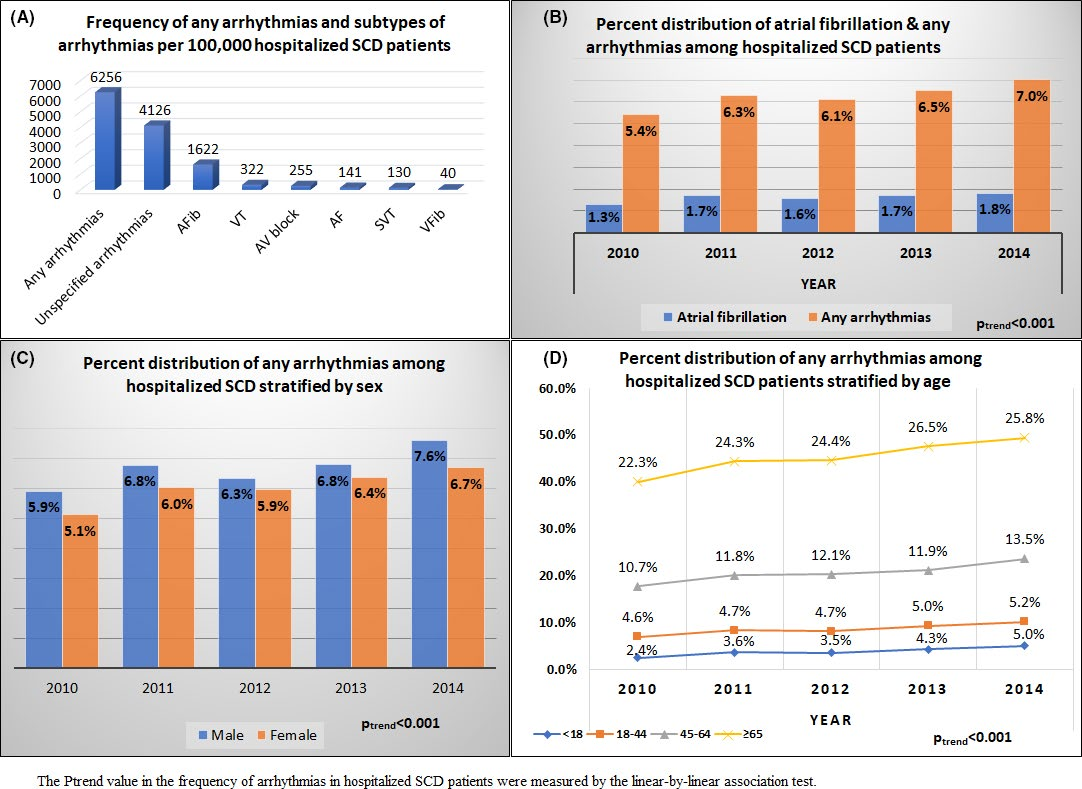
(A) Frequency of any arrhythmias and subtype of arrhythmias per 100 000 SCD‐related hospitalizations. (B) Percent distribution of any arrhythmias and atrial fibrillation among SCD‐related hospitalizations. (C) Percent distribution of arrhythmias among SCD‐related hospitalizations stratified by gender. (D) Percent distribution of arrhythmias among SCD‐related hospitalizations stratified by age

The frequencies of arrhythmias documented per 100 000 hospitalized SCD patients were as follows: any arrhythmias (6256 per 100 000), unspecified arrhythmias (4126 per 100 000), AFib (1622 per 100 000), AF (141 per 100 000), SVT (130 per 100 000), atrioventricular (AV) block (255 per 100 000), VFib (40 per 100 000) and VT (322 per 100 000) (Figure 1A). The frequency of supraventricular arrhythmia (AFib, SVT, and AF) and ventricular arrhythmia (VFib and VT) were 1893 and 362 per 100 000 SCD‐related admissions, respectively. The frequency of any arrhythmia in hospitalized SCD patients increased from 5.4% in 2010 to 7.0% in 2014 (29.6% relative increase, *P*_trend_ < .001). The frequency of AFib increased from 1.3% in 2010 to 1.8% in 2014 (38.5% relative increase, *P*_trend_ < .001) among hospitalized SCD patients (Figure 1B). From 2010 to 2014, the frequency of arrhythmias in hospitalized male and female SCD patients relatively increased by 28.8% and 31.4%, respectively (Figure 1C) (*P*_trend_ < .001). Among hospitalized SCD patients, the frequency of arrhythmia increased nearly two‐fold (108% relative increase, 2.4% in 2010 to 5.0% in 2014) in <18 years old age group (*P*_trend_ < .001). The frequency of arrhythmias among hospitalized SCD patients increased by 13.0% in those aged 18‐44, 26.2% in those aged 45‐64 and 15.7% in those aged ≥65 during the study period (Figure 1D).

### 3.3 Odds of in‐hospital mortality in SCD‐related hospitalizations with arrhythmias

The SCD cohort with arrhythmia demonstrated a higher odd of in‐patient mortality (unadjusted OR 6.81, 95% CI 5.95‐7.80, *P* < .001; adjusted OR 2.53, 95% CI 2.15‐2.97, *P* < .001) as compared to SCD patients without arrhythmia.

## 4 DISCUSSION

This study reveals a significant increase in the frequency of arrhythmias in hospitalized SCD patients over the study period. Unspecified arrhythmias (65.9%) were seen most frequently, followed by AFib (25.9%) among SCD‐related admissions. The frequency of arrhythmias among hospitalized SCD patients increased in all age and gender groups. However, the relative increase in the frequency of arrhythmias in hospitalized SCD patients was most pronounced in females and patients younger than 18 years. Another notable finding of the study was that the arrhythmia incidence during hospitalizations for SCD was a significant predictor for worse outcomes, including a higher prevalence and odds of all‐cause mortality, prolonged length of stay and higher total hospital charges as compared to the cohort without arrhythmias.

Nearly three decades ago, Maisel et al reported that nearly 80% of SCD patients experienced cardiac arrhythmias during vaso‐occlusive pain crises.^8^ The reason for the increased frequency of arrhythmias in SCD patients remains largely unclear, but a predilection to cardiac autonomic dysfunction, QT prolongation, and myocardial fibrosis have been posited as possible explanations.^15^, ^16^ Indik and colleagues studied two cohorts of SCD and established that a prolonged QT interval and ventricular tachyarrhythmias were significant predictors of mortality in this population.^17^ We observed a nearly threefold higher odds of in‐hospital mortality among SCD hospitalized patients with arrhythmias as compared to those without arrhythmias on multivariate analysis. In keeping with this finding was a study that suggested that a prolonged QT was an independent predictor of increased sudden deaths in patients with SCD.^18^ Moreover, Gacon et al revealed that AV block during the vaso‐occlusive crisis due to an ischemic event at AV node and bundle of hiss could be contributory to abnormal cardiac rhythms in SCD.^19^ As increasing utilization of telemetry services during hospitalizations, overall rising trends of arrhythmia irrespective of underlying medical conditions were observed throughout time.^20^ Our study also showed rising trends in arrhythmias among SCD cohort between 2010 and 2014. It is well‐established that traditional risk factors such as hypertension, diabetes mellitus, congestive heart failure, chronic pulmonary disease and behavioral issues such as drug/alcohol abuse are major risk factors for developing cardiac arrhythmias in the general population and our cohort also showed higher burden of all these comorbidities among SCD cohort with arrhythmia which could be the confounding factors in hospitalization outcomes.^12^, ^21^ However, our study showed that SCD cohort with arrhythmia raised the odd of in‐patient mortality in the adjusted multivariate regression model. Arrhythmias in SCD patients may lead to a greater number of in‐hospital complications and necessitate increased diagnostic workup, factors which may account for the observed increase in hospital length of stay in this cohort. This result suggests that early recognition and prompt treatment of cardiac arrhythmias using telemetry services for hospitalized SCD may be helpful in lowering the burden of sudden cardiac death in SCD patients; further research is warranted to explore this possibility. It is also possible that the association between arrhythmias and increased mortality in this population is not causative; the presence of arrhythmias may simply serve as a marker of advanced disease in patients with SCD.

## 5 LIMITATIONS

Limitations of this retrospective analysis are largely related to the limitations of NIS data interpretation. Diagnoses may have been coded incorrectly during patient encounters due to human error. Due to the anonymized nature of NIS data, the frequency of arrhythmias in hospitalized SCD patients may have been overestimated if individual patients presented for multiple inpatient encounters (eg, an individual patient cannot be followed longitudinally in NIS data). The cause of death cannot be adjudicated from NIS data. Propensity‐matched analysis was not performed in this study which could be another limitation in assessing the outcomes between two cohorts. Nevertheless, this is the largest nationally representative study exploring the burden of arrhythmias and their role as predictors of worse outcomes in hospitalized SCD patients.

## 6 CONCLUSIONS

The frequency of arrhythmias among hospitalized SCD patients is on the rise, unspecified arrhythmias were seen most frequently followed by AFib among SCD‐related admissions. The presence of arrhythmia in SCD was associated with worse in‐hospital outcomes and increased healthcare resource utilization. Further research is required to explore whether or not early recognition and management of arrhythmias in the SCD population will lead to improved clinical outcomes.

## CONFLICT OF INTEREST

None.

## Supporting information

Table S1
