# Supplementary material for: Sickle cell disease‐associated arrhythmias and in‐hospital outcomes: Insights from the National Inpatient Sample
Source: J Arrhythm. 2020 Aug 8;36(6):1068–73. doi: 10.1002/joa3.12418 (PMC7733582; doi:10.1002/joa3.12418)
Supplement: Supplementary file 1 — Table S1 [file JOA3-36-1068-s001.docx]

| Supplementary Table. Odds of in-hospital mortality in SCD-related hospitalizations | | | | |
| --- | --- | --- | --- | --- |
| Variables | **Adjusted Odds Ratio** | **95% C.I.** | | **P-value*** |
|  |  | **LL** | **UL** |  |
| Age | 1.02 | 1.01 | 1.03 | <0.001 |
| Male vs. Female | 1.08 | 0.86 | 1.36 | 0.492 |
| Race |  |  |  |  |
| White vs. Other | 0.91 | 0.39 | 2.10 | 0.817 |
| African American vs. Other | 0.81 | 0.41 | 1.52 | 0.539 |
| Hispanic vs. Other | 0.98 | 0.41 | 2.39 | 0.969 |
| Asian or Pacific Islander vs. Other | 0.93 | 0.36 | 1.71 | 0.993 |
| Native American vs. Other | 2.92 | 0.53 | 4.38 | 0.215 |
| Weekday vs. Weekend admission | 0.95 | 0.73 | 1.23 | 0.685 |
| Primary expected payer |  | | |  |
| Medicare vs. Others | 1.10 | 0.54 | 2.24 | 0.787 |
| Medicaid vs. Others | 0.71 | 0.35 | 1.45 | 0.347 |
| Private including HMO vs. Others | 1.0 | 0.52 | 2.19 | 0.858 |
| Self-pay vs. Others | 0.96 | 0.41 | 2.22 | 0.924 |
| No charge vs. Others | 1.22 | 0.25 | 5.94 | 0.805 |
| Location teaching status of hospital |  |  |  |  |
| Rural vs. Urban teaching | 0.92 | 0.43 | 1.94 | 0.824 |
| Urban nonteaching vs. Urban teaching | 0.84 | 0.50 | 1.40 | 0.502 |
| Hospital region |  |  |  |  |
| Northeast vs. West | 0.974 | 0.597 | 1.591 | 0.917 |
| Midwest vs. West | 0.890 | 0.530 | 1.494 | 0.659 |
| South vs. West | 1.240 | 0.816 | 1.886 | 0.313 |
| Bed size of Hospital |  |  |  |  |
| Small vs. Large | 0.66 | 0.42 | 1.05 | 0.082 |
| Medium vs. Large | 0.96 | 0.74 | 1.27 | 0.793 |
| Comorbidities |  |  |  |  |
| *Alcohol abuse* | 1.96 | 1.14 | 3.37 | 0.015 |
| *Deficiency anemias* | 0.83 | 0.61 | 1.12 | 0.218 |
| *Congestive heart failure* | 1.98 | 1.45 | 2.68 | <0.001 |
| *Chronic pulmonary disease* | 0.91 | 0.67 | 1.22 | 0.522 |
| *Coagulopathy* | 4.64 | 3.57 | 6.03 | <0.001 |
| *Depression* | 0.50 | 0.30 | 0.84 | 0.009 |
| *Diabetes, uncomplicated* | 1.07 | 0.73 | 1.56 | 0.736 |
| *Diabetes, chronic complications* | 1.35 | 0.79 | 2.29 | 0.260 |
| *Drug abuse* | 0.57 | 0.34 | 0.96 | 0.034 |
| *Hypertension* | 0.93 | 0.71 | 1.21 | 0.591 |
| *Hypothyroidism* | 0.92 | 0.55 | 1.54 | 0.749 |
| *Liver disease* | 1.91 | 1.31 | 2.78 | 0.001 |
| *Fluid and Electrolytes disorders* | 2.26 | 1.79 | 2.85 | <0.001 |
| *Obesity* | 0.76 | 0.47 | 1.21 | 0.245 |
| *Paralysis* | 1.04 | 0.56 | 1.93 | 0.910 |
| *Peripheral vascular disorders* | 1.51 | 0.89 | 2.54 | 0.120 |
| *Psychoses* | 0.59 | 0.32 | 1.09 | 0.091 |
| *Pulmonary circulation disorders* | 2.21 | 1.61 | 3.03 | <0.001 |
| *Renal failure* | 1.22 | 0.90 | 1.65 | 0.201 |
| *Valvular disease* | 0.76 | 0.47 | 1.24 | 0.272 |
| *Weight loss* | 1.88 | 1.29 | 2.75 | 0.001 |
| *Sepsis* | 5.86 | 4.56 | 7.53 | <0.001 |
| *Acute myocardial infarction* | 2.64 | 1.38 | 5.04 | 0.003 |
| *Acute chest syndrome* | 1.07 | 0.66 | 1.73 | 0.790 |
| *Pneumonia* | 1.44 | 1.09 | 1.90 | 0.011 |
| *SCD with crisis* | 0.66 | 0.51 | 0.86 | 0.002 |
| *Splenic sequestration* | 0.98 | 0.39 | 2.40 | 0.960 |
| *Arrhythmia* | **2.53** | **2.15** | **2.97** | **<0.001** |
| Multivariable predictors of In-patient Mortality in sickle cell disease (SCD) hospitalizations. Note: Multivariate regression model was adjusted for baseline demographics including age, sex and race, payer status, admission day (weekend or weekdays), hospital bed size, location/teaching status and region, all baseline comorbidities. The SCD cohort with arrhythmia demonstrated a higher odd of in-patient mortality (adjusted OR 2.53, 95% CI 2.15-2.97, p<0.001) as compared to SCD patients without arrhythmia.  HMO=health maintenance organization, CI=confidence interval, LL=lower level, UL=upper level, *P<0.05 indicates clinical significance. | | | | |
